# ‘Time’ For ‘The People’. Reflections on ‘Psychoanalysis For The People: Free Clinics and The Social Mission of Psychoanalysis In Our Times’

**DOI:** 10.3366/pah.2022.0445

**Published:** 2022-12

**Authors:** Lisa Baraitser

**Affiliations:** University of London, London, United Kingdom

**Keywords:** care, emancipation, temporality, revolutionary time, repetition

## Abstract

In this paper I offer some reflections on two important conferences held at the Freud Museum in London during 2021, which has resulted in the publication of a remarkable special issue of *Psychoanalysis and History*. The conferences aimed at providing a new space to re-engage a long history of debate, started by Freud himself, about psychoanalysis as not only a form of mental health treatment, and a theory of mind, but a social and political project aimed at emancipation. Descriptions of pioneering ‘social clinics’ from São Paulo to South London that maintain psychoanalytic thinking about social suffering and offer psychoanalysis as a critical analytic tool to understand such suffering, render these projects ‘psychosocial’. I reflect on the temporal nature of these clinics – their particular uses of time as part of healing, as well as their temporariness that is linked to the precarity of projects that are often underfunded, and rely on the passion and commitment of founders, practitioners, and patients. Somehow many of them ‘stagger on’, contributing to the preservation of the social mission of psychoanalysis, started over 100 years ago. I offer a perspective from the ‘Waiting Times’ research project, that investigates the relation between time and care, by turning to Isabelle Stengers’s ‘care of the possible’ as a way to conceptualise the work of these psychoanalytic social clinics.

## Introduction

In 2021, two important conferences were held at the Freud Museum, London, titled ‘Psychoanalysis for the People: Free clinics and the social mission of psychoanalysis in our times’. They emerged out of discussions between the psychoanalyst and academic Raluca Soreanu, who was at the time part of the research team of the *Waiting Times* project, a Wellcome Trust funded research initiative exploring the relation between time and care^1^; the psychoanalytic psychotherapist and author Joanna Ryan who has written extensively on the politics of psychoanalysis; and Ivan Ward, Deputy Director and Head of Learning at the Freud Museum. They aimed to provide a space to reengage and rejuvenate a long history of debate, started by Freud himself, about psychoanalysis as not only a form of mental health treatment, and a theory of mind, but a social and political project aimed at emancipation as much as the relief of suffering, and at instigating changes to social and political life worlds as much as psychical ones. These turned out to be landmark events. Taking place in the middle of the Covid-19 pandemic that highlighted the entrenchment of structural inequalities and the emergence of new forms of vulnerability and interdependency, it was a timely, if painful, moment to draw together diverse perspectives on what we might mean by psychoanalysis ‘for the people’. Who, for instance, are ‘the people’ who may need or benefit from psychoanalytic treatment, especially in the midst of an epidemic of loneliness and a growing world-wide mental health crisis in post-pandemic times, and who has access to such treatment? What is the relation between psychoanalysis as a project of emancipation, and how the category of ‘the people’ is formed, governed, and maintained, especially how psychoanalysis may collude with, as well as intervene in, the norms of such categorisation? With populations still locked down in collective practices of ‘waiting’ in the name of protecting health services and shielding those deemed vulnerable to the virus, whilst simultaneously exposing others to the brutalities of front-line work without protection, to domestic violence in the home, the precariousness of homelessness, or the particular dangers of lockdown in conditions of incarceration, how can the ‘waiting’ entailed in psychoanalytic practice help us think about the relation between care and violence? And what can psychoanalysis, as a form of social and political critique, offer to pressing concerns about equality, freedom, interdependency, care, and social and political change?

One of the most important things to emerge from the conferences, and this subsequent special issue, is the visibility of numerous projects worldwide, some longstanding, others more temporary and fragile, from São Paulo to South London, all of which seek to offer a form of ‘mutual aid’ that we saw resurface during the pandemic, as well as to preserve the work of psychoanalysis despite the constant pressure to devalue its currency – that of a particular orientation towards time and truth – as it emerges from ‘the people’ and for ‘the people’. Many are grass roots organizations that refuse the distinction between those offering and those using the service, and locate the service as belonging to those who use and need it. In this sense psychoanalysis simply *is* the people who use it. This holds for all of the projects described here: Baffour Ababio’s account of Nafsiyat, the long-standing intercultural therapy centre established in London in 1983 by Jafar Kareem; the Psychosis Therapy Project founded by Dorothée Bonnigal-Katz; the Refugee Therapy Centre outlined by Aida Alayarian; Usemi Racial Trauma Clinic discussed by Earl Pennycooke; the Battersea Aid and Action Centre and its development of ‘social action psychotherapy’ inspired by the work of Sue Holland and Julian Lousada in South London in the 1970s; Kristina Valendinova, Antoine Huon, and Xavier Fourtou’s more recent South London project, Bubble and Speak, inspired by Françoise Dolto’s Maison Vertes; the almost 100-year old London Clinic of Psychoanalysis, critically reflected on here by Penny Crick; and Kwame Yonatan Poli dos Santos’ compelling description of the work of *Margens Clínicas* in Brazil. These projects are themselves just a tiny sample of hundreds of psychoanalytic centres around the world that attempt to respond to the question of mental and social pain by organizing an alternative set of relations between care, time and money that aims at democratizing psychoanalysis as it also sustains it. What becomes clear is how these ‘social clinics’ (as some of them refer to themselves) continue to maintain and protect a mode of thinking and practice that is, at its best, anti-normative, and works against the grain of most prevailing approaches to mental health treatment, and the human subject. In insisting that this work is a collective and shared endeavour, the clinics reveal that psychoanalysis is propped up by ‘the people’ as much as it supports ‘the people’, a form of mutual aid, we could say, between psychoanalysis and the people. Somehow, through this collective commitment, and often the enormous passion and vision of those involved, an insistence that psychoanalysis has something to offer the social and political sphere is maintained.

In what follows I reflect on the relations between psychoanalytic time, social violence, and care, that I think each project raises in distinctive ways. I offer some initial reflections on what the offer of psychoanalytic time for something called ‘the people’ might mean, especially as social violence is not only ‘out there’ but also situated internally to psychoanalytic theory and practice. I draw on Raluca Soreanu’s two invaluable concepts of ‘psychoanalytic convertibility’ and ‘friction’ to re-orientate this relation between psychoanalysis and ‘the people’. Here the idea of forming new relations between care, violence, and time are elaborated, as well as the way that psychoanalytic time itself persists as anachronistic within the history of psychoanalysis. I relate this to the work of the *Waiting Times* research project, drawing on Isabelle Stengers’ notion of ‘care of the possible’ alongside Kwame Yonatan’s articulation here of ‘*aquilombamento nas margins’*, a form of anti-racist work that draws on the Brazilian term ‘quilombo’, a world without colonial walls, to help us understand the simultaneous work of preservation and critique that is entailed in the social mission of psychoanalysis.

## ‘Time’ for ‘The People’

When is it ‘time’ for ‘the people’? When Freud made his pledge of psychoanalytic time to the ‘poor man’ in his speech to the Fifth International Psychoanalytic Congress in Budapest in 1918, it was futural, posited as a promise – a ‘gift’ in the Derridean sense – that could be glimpsed only in the yet-to-come:

[…] it is possible to foresee that at some time or other the conscience of society will awake and remind it that the poor man should have just as much right to assistance for his mind as he now has to the life-saving help offered by surgery […] (Freud, 1918, 17, p. 167)

Psychoanalytic time would be freely given ‘at some time or other’, a time supplementary to ‘now’, when the conscience of society would awake. However, as the projects described here attest, psychoanalytic social clinics deliberately grasp this time, insisting, with innovation, determination, and tenacity, that it happens ‘now’. In this psychoanalytic mode, there is only ‘now’, as the supplementary time of the conscience of society is always immanent in historical time; ‘now’ is always already the time for ‘the people’.

Perhaps the question then is not ‘when’ but ‘what’ is this time for ‘the people’, evoked by Freud’s notion of ‘the poor man’? Time, as Fanny Söderbäck tells us in her thesis on revolutionary time, is always an issue of power, just as much as power underpins any notion of the demos or people. Queer, crip,^2^ and decolonial critiques of models of time that structure the global temporalities of the present reveal that the normative temporal regimes that reproduce the rhythms of sociality are patriarchal, colonial, ableist, and hetero- and cis-normative (Söderback, 2019, p. 15). As many scholars of social time have pointed out, they discipline and marginalize those who fail to conform to the temporal conventions of western modernity, especially the norms of progress and development, and the patterned ways that time is supposed to sequentially unfold through birth, growth, maturation, reproduction, the accumulation of resources and wealth, its passing on to the next generation, and death (Luciano, 2007; Chakrabarty, 2009; Freeman, 2011, 2019; Kafer 2013). As Elizabeth Freeman puts it:

From the tuning of bodies toward maternal, familial, and cultural rhythms, through the rise of national feeling across geographic borders, through the standardization of calendars and clocks, through the factory system of production and the aesthetics that accompanied it, synchrony *creates* the social. (Freeman, 2016, p. 133, original emphases)

Furthermore, although Freeman notes that from an anthropological perspective subjects become legible by synchronizing first with their family’s daily rhythms, and then with their culture’s events and rituals that lay out a trajectory for a meaningful life, in the broader historical perspective of the modern period (from industrialization in Europe during the mid-1700s to the present) it is through the emergence of institutionalized techniques of biography – of narrating and documenting one’s story in particular ways – that protection or care is granted or withheld. These techniques include the rise in the late nineteenth century of the psychoanalytic case study, the parole hearing, and the petition from asylum. From this perspective not only synchrony but also anachrony – time that is revealed as ‘wrong’, that fails to narrate in certain ways – creates a caring or uncaring social world (Freeman, 2016, p. 130).

This means that psychoanalysis is already caught up with practices of inclusion and exclusion that decide who can gain access to protection and care. Those who cannot narrate their lives in accordance with normative developmental timelines show up in the social fabric as ‘antisocial’; ‘behind’ the contemporary, ‘arrested’ in their development, ‘backwards’, and stuck. In particular, what Charles Mills calls ‘white time’ sets the pace and tempo of modernity so that non-white time is *always* ‘wrong’ (Mills, 2014, 2020). This is underpinned, as Aníbal Quijano famously described, by the ongoing and ever-present effects of colonisation that he calls ‘coloniality’ in which the colonial past lives on in the present, structuring all human relations (Quijano, 2010). The work of decoloniality is the uncoupling of modernity from white time that emancipates the multiple times of cultures and civilizations upon which the Western imperial project imposes its conceptualization of time (Mignolo, 2018). Without this uncoupling, ‘time’ and ‘the people’ go on mutually reproducing one another: models of time give rise to norms and the everyday practices that synchronize them, norms that structure who gets to be included in ‘the people’; and, in turn, those who are admitted into the category of ‘the people’ reproduce Western models of time that are naturalized by those whom they privilege. Psychoanalysis is deeply imbricated in this process.

To interrupt this stagnant cycle between ‘time’ and ‘the people’, Söderbäck calls for ‘revolutionary time’ (Söderbäck, 2019). Linking the broader process of decolonisation with histories of feminist thought that seek to intervene in the classical delineation between cyclical time (women’s time, the time of nature, reproduction, and the regeneration of the species) and linear time (time’s masculine teleological trajectories that drive towards progress, linked to the nation state and its apparatuses of violence), revolutionary time is a movement of perpetual return and renewal. Söderbäck is keen, however, to distinguish the temporalities of return and renewal from those of repetition. In Söderbäck’s analysis, cyclical and linear-progressive time are both caught up with repetition. Cyclical time by definition repeats itself indefinitely; and linear-progressive time is driven by an ideological desire to produce a future according to already established ideals and narratives – more of the capitalist same. Given that cyclical and linear time map on to the gendered distinctions between nature-woman-immanence and culture-man-transcendence, Söderbäck’s intervention is to construct a revolutionary model of time and transcendence that neither confines women, and others who may fall out of normative notions of ‘the people’, to the realm of embodiment, nor represses the body altogether. Instead, it offers a model of time which recognizes embodiment as the condition of possibility for futurity. If we take embodiment to mean the disruptive and inchoate drives that animate the symbolic markers of difference that operate, albeit differentially, for all bodies, then futurity from the perspective of revolutionary time does not simply repeat more of the same, but opens up the chance of futurity itself: Mignolo’s multiple times of the many bodies that western modernity supresses, and with it the possibility of something different, of change. ‘Time’ for ‘the people’, then, is not simply synonymous with Freud’s offer of psychoanalytic time to the ‘poor man’. Instead, it is the time that can break open the repetitions of cyclicality and teleology, the revolutionary time of both psychic and social emancipation.

All of the psychoanalytic clinics described here offer an articulation of the revolutionary time of emancipation, showing not just what is aimed for in a future when social conscience is finally wakened, but what can be done within historical time to break open the repetitions of cyclicality and teleology as they play out in everyday psychosocial life. Indeed, many of the projects start from the premise that emancipation is only possible when both psychic and social change are synchronous, when both are activated in or ‘with’ time, as the etymological meaning of synchrony points towards, even if there are lags in pace and points of departure. Earl Pennycooke, for instance, identifies the traumas of anti-Black racism that are levelled at Black men as the overwhelming of Black masculinity by Thanatos. This is a death drive that emanates not just from within but from those social structures that produce Black masculinity as supposedly ‘dangerous’ or ‘exciting’, something to be contained or feared, and ultimately destroyed. He points us towards the psychic effects of the grinding micro-violences of ‘endemic exclusions and discriminations’, as Dorothée Bonnigal-Katz puts it, in her description of the work of the Psychosis Therapy Project. But by working psychoanalytically, there is a refusal of a neat distinction between inside and outside, with the psyche ‘in here’ and the social ‘out there’. Instead, these projects foreground a psychoanalytic formulation of the social mind that I would call ‘psychosocial’. To work psychoanalytically whilst engaging synchronously with what Kwame Yonatan evocatively calls the ‘pathologies of social structure’, is to upset an idea of the outside as an already established social structure that presses in on the subject, or the inside as an already established psychic structure that meets that constituted world. Freud, as Patricia Gherovici reminds us in her important contribution on racisms, exclusion, and universalism, offers an account of the ego as a psychic entity that comes into being with and through hatred: hatred of what are simultaneously felt to be internal needs, and perceived (and at times actual) hostile external objects. It is hate that comes to differentiate the boundary between inside and outside, ensuring its permanence as its constituting principle. Judith Butler, in *The Psychic Life of Power*, shows how the mechanism of this boundary formation occurs through the ego’s melancholic incorporation of social norms – violent norms, including racism and the norms of whiteness, that become part of the very structure of the ego through the difficulties of loss and mourning (Butler, 1996). Internal objects are built on relations with external objects that are already imbued with discourses of patriarchy, imperialism, racism, nationalism, sexism, ablism, and class, so that we never simply love and hate our objects but are involved in a ‘relational passion’ as Amy Allen puts it, whereby love and hate are two competing modes of *social* relatedness that constitute the border between psychic and social life (Allen, 2021, p. 39).

The projects described here therefore attest to ways that psychoanalysis’ social mission engages precisely with the ego at the level of its psychosocial formation. Each project understands in its distinctive ways that the social work of psychoanalysis is certainly to find innovative ways to ‘common’ psychoanalysis, making it more accessible and affordable for those that need it. But more crucially, perhaps, it aims to intervene at the level of the ego’s formation itself, where the boundary between social and psychic life is open to both violation and repair. It is here that the possibilities of freedom lie. It is in our capacities to know about our openness to both violence and care, and in knowing about this, to mourn violations and enact self-repair, that psychoanalysis can provide the psychic conditions for changing the social world. Yonatan, for instance, describes the work of the *Margens Clínicas* group, a collective of ten psychoanalysts and psychologists in Brazil, as dedicated to thinking about the interfaces of psychological suffering with the pathologies of social structure, using what he calls ‘clinical listening’ to confront state violence so as to restore the value of a life that has been the object of such violence. Distinct from both empathy and sympathy, here clinical listening that may give rise to mourning and repair entails attending to unconscious phantasy and its vicissitudes, as the ego attempts to deal both with its hate and its hate-filled objects. This work breaks open the repetitions of cyclicality and teleology as they play out in everyday psychosocial life, bringing revolutionary time into the consulting room.

## A New Economy of Listening

Raluca Soreanu, with colleagues Tereza Mendonça Estarque and Maria de Fátima Lobo Amin from the Institute of Complexity Studies in Rio de Janeiro, help us understand the economy of such listening through her invaluable concept of ‘psychoanalytic convertibility’. She describes this as an alternative economy of time, money and suffering. One such alternative economy was developed at the Institute through a system called *caixa único* (or ‘single pot’). The single pot distributes three elements: the funds brought in from patients and associates; the analyst’s time; and a certain willingness to belong to a group or collectivity that is itself put into circulation, and that Soreanu, Estarque, and Amin link to a *spontaneous engagement in the service of others*. Because the Institute sets the average value of the analytic hour based on what money has been collected in any particular month from the patients who have attended, the hourly fee (the link between time and money) is established by the collective or institution rather than the individual. No individual analyst charges a specific fee, and no individual patient is identified as one who pays a specific fee. The fee is collectivized in such a way that it puts the commitment to the *shared* work of psychoanalysis, shared listening, itself into circulation; two terms (time and money) become three. This overflowing of a spontaneous and effusive element that is more than time and money constellates the other two terms, opening what Soreanu calls a ‘local psychoanalytic currency’ – one that is negotiated in different ways by each psychoanalytic social clinic. These clinics attest to the passionate and creative work of running, in Soreanu’s terms, their own alternative and anti-capitalist ‘money’. This is not only about eradicating payment, or lowering the fee, but about putting the collective or shared commitment to clinical listening into circulation in such a way as to produce alternative forms of conversion between time, money and suffering. In doing so, these collectives and institutions enable the long repetitive process of psychoanalytic working-through to be reconfigured as it makes contact with the local conditions in which people live their lives, often in what Elizabeth Povinelli calls ‘the seams of capitalism’ (Povinelli, 2011). When clinical listening, understood in this sense of a spontaneous and effusive commitment to the suffering of others, is put into circulation in the social body, then the chance of a future may emerge, indeed both for psychoanalysis as well as ‘the people’. It dislodges both the linear time of departure and arrival, and the chronic repetitions of the systemic violence of marginalization, exclusion, and discrimination – the choking of life-chances and of the value of life itself.

It is not just synchrony between psychic and social worlds, then, that a social psychoanalysis may offer. The temporality of clinical listening overflows linear time, but shows up within western capitalist time as anachrony – ‘wrong time’ – and it is this that is at the heart of its revolutionary potential. This is what Julia Kristeva calls a ‘scandal’ (Kristeva, 2003) that underpins what she identifies as a more generalized cultural resistance to psychoanalysis, and its ambivalent place in both culture and perhaps in current mainstream mental health treatment which is now largely driven by ‘big pharma’ and the cognitive turn in psychology. Freud, as André Green elaborates in *Time in Psychoanalysis* (Green 2002), offers a series of interlinked models of temporality that move sequentially within his own thought. It starts with the time frozen or blocked by fixation and trauma in ‘Studies On Hysteria’ in 1895, in which memory radiates out in what André Green calls ‘transchronic’ ways. This gives way to Freud’s notion of *nachträglichkeit*, as it appears in ‘A Project for a Scientific Psychology’, in which the ever-presentness of unconscious life is understood as the constant temporality that accompanies waking life and not just dream time or psychopathology, a permanent temporality which dismembers time, as Green puts it, and splinters it into non-uniform and bidirectional forms, with infantile sexuality and its amnesia as its driving force. The individual’s prehistory is given central place in psychic formation that is formed retroactively through the double-time of afterwardness, especially through what the child does with the enigmas of sexuality in the present-tense of the child-adult situation that Jean Laplanche draws our attention to, and relates to the question of origins and psychic binding that brings the temporality of the adult’s unbound sexuality into play. There is Freud’s later elaboration of the building up of the ego through melancholic attachments to lost objects whereby the ego is established through a failure to face mourning, with the end of the object’s existence taken back into the beginnings of the ego’s psychic life. This means the ego is permanently sutured to the past without being aware of its own structuring in relation to this un-lost object. There is phylogenesis and the recapitulations and repetitions of primal scenes; the insistent rhythms of repeating, remembering, and working through; and eventually the temporality of the death drive itself, the perpetual arc of temporal return which traces the contours of the detour that will have been a life.

For Kristeva, however, the scandal at work in Freud’s unfolding temporal model is the break with both biological time and the time of consciousness. Unconscious time – the element of the psyche that is unbounded by time – is ‘wrong’ in the sense of ‘unnatural’ or ‘against’ the biological, which is the time of development, maturation, and decay. The refusal of the unconscious to accept the passing of time means that anachrony constantly disrupts and gives the lie to synchrony. André Green locates this scandal in the fact that the unconscious refuses to reflexively know about the passing of time, about its repetitions, movements, developments, loops and its dissolution. This unbound time is not strictly negativity but it is revolutionary in maintaining its own time within historical time. Elizabeth Freeman (2016) helps to historicize this, writing that modernity invents anachrony as ‘the fundamental condition of modern subjectivity. The name for it was the unconscious […]. Freud recognized anachrony as the condition from which moderns suffered and through which modern subjects emerged’ (p. 136). In modern times the psyche operates outside the laws of progressive time. In the extreme, timelessness or *Zeitlosigkeit* is the time of death; but psychic time is not dead time. Rather, it has its *own* time, an atemporal time; it is ‘a matter of a detained temporality, a temporality that does not temporalize, a breach of a time that does not temporalize’ (Kristeva, 2003, p. 31).

The effusive commitment to the suffering of others that manifests as clinical listening engages the atemporal yet revolutionary time of the unconscious and links with the second concept Soreanu offers us, which she calls ‘friction’.^3^ Friction describes a relation that a semi-autonomous collective of psychoanalytic practitioners may take up in relation to the demands of the state; policies, for instance, that undermine the mental health of citizens whilst extracting their labour and promoting the internalization of the state’s responsibilities as forms of ‘self-care’. Friction would describe an ‘up-againstness’, Soreanu states, that is not so much a retrenchment from the state, or simply an antagonistic relationship to state institutions, but rather a practice of using friction’s force as a source of creativity and innovation to develop services that can intervene in and mitigate state violence and its failures to care. Soreanu develops a temporal analysis of friction, claiming that being in friction with state agendas is also to be de-synchronised from them, to work at a different pace, rhythm, and tempo from the state’s beat. This does not place a psychoanalytic community outside of historical time but, rather, produces a psychoanalytic community through the collective practice of insisting on a *different* time, the anachronistic time of the unconscious, within the state’s time.

Baffour Ababio, in his paper describing the history of Nafsiyat, for instance, notes that Jafar Kareem who founded the centre in the 1980s, hoped for a time when the service would become redundant, when the principles of the practice developed in the clinic would have been integrated into mainstream analytic practice. These include the engagement of the whole being in the therapeutic relationship, the full acknowledgement of the role of intergenerationally transmitted historical traumas of racism, and of the multi-layered and constantly interacting relation between identities and social positions. Ababio notes some progress, some improvement within the psychoanalytic profession that has occurred over 30 years. But perhaps more prominent is the undertow that undercuts such progress within a profession that remains stubbornly white, alongside the cultural drag that means that, as Ababio elucidates, the promise of progress in race relations that emerged from the Macpherson report in the UK in 2000, for instance, has failed to root out institutional racism in the Metropolitan Police even in 2022. Racism has to be raised again and again, as if for the first time, just as a seminar series on psychoanalysis’ social mission that took place at the Freud Museum in 1993, entitled ‘Psychotherapy Black and White’, has to be produced again thirty years later. Dorothy Holmes has recently reflected on the many published papers that she has produced over the last fifteen years that have suggested that psychoanalysis and related disciplines ‘have gone along with the general societal trend to disown the destructiveness of racism in its manifold forms, affecting all of our lives, and in its deep psychic structures and mechanisms such as disavowal’ (Holmes, 2021, p. 240). The creative practice of insisting on a different time, is also the political work within psychoanalysis of going on saying the same things, again and again.

## Repetition as a Practice of Care

The time of ‘again and again’ is the time of repetition. Despite Söderbäck’s positioning of revolutionary time as distinct from the dead time of repetition, I would argue that a psychoanalytic reading of repetition helps us to understand the temporalities of care that I think are at work in the act of clinical listening that we have been discussing here. In recent work I have elaborated an account of a ‘maternal death drive’ that supplements Freud’s death drive, sustaining and supporting a relationship to ‘life’ as a form of ongoing time, but remaining ‘otherwise’ to the life drive or pleasure principle, and therefore imbedded within the death drive proper (Baraitser, 2017, 2020). Adrian Johnston has shown how the drives do not operate according to one monolithic temporality but are simultaneously timeless and temporal, taking the form of both iteration (repetition, which accounts for the constancy of the death drive) and alteration (development or change, which accounts for the capacity for the drive to change object and aim) (Johnson, 2005). Although repetition can be understood as a way of negating life by ‘restor[ing] an earlier state of things’, as Freud puts it in *Beyond the Pleasure Principle* (Freud, 1920), repetition, as the long history of feminist thought attests to, also has to do with practices of maintenance, perseverance, and endurance that work to preserve and prolong life. I use the term ‘maternal’ in conjunction with the death drive to indicate a figure who is not necessarily linked to biological femininity and birthing, but nevertheless signifies a relational element that enables the unfolding of another life in relation to one’s own path towards death, and marks the point that alteration and iteration cross one another. Although we do die our own death, there are practices of permanent labouring that are bound to repetition and animate ‘life’ in such a way as to support and allow the subject to die in its own way. This maternal death drive is ‘passive’ to the extent that it entails a form of inactivity, a capacity to wait for the other to unfold. Crucially, this repetition that sustains something unfolding after its own fashion is a form of labour that is not a matter of indifference to the labouring subject; instead, maternity, in its failure to be indifferent to the specificity of its labour, implies a return, again and again, to a scene of mattering. Here the time of repetition that involves maintenance and repair, interrupts the temporalities of productivity. Instead, repetition signals the time it takes for lives to come to matter to one another even as they die their own deaths. These, then, are forms of care that take time but also make time, producing pockets of time’s flow within the otherwise static temporal horizons in which we wait together for the unfolding of disasters – the disasters of climate catastrophe, mass extinction, war, state repression, resource extraction, overconsumption – that are already immanent in the present.

We could think about ‘free’ psychoanalysis as one such pocket. The fee, after all, pays for the psychoanalysts’ time, but not their thoughts, attention, skill, craft, or soul. These are offered over and beyond the fee as a practice or form of waiting without an outcome in mind, a waiting that is not remunerated through the mechanism of the wage, but through time itself. We know that the economies that have structured social relations since the 15^th^ century – what we loosely call capitalism – rely on ‘somebody’, that is, some bodies to pay for the free circulation of capital. Historically, Joy James has argued, these are Black enslaved bodies, especially the maternal body that she names the ‘Maternal Captive’ that continues to prop up all forms of capitalist production today (James, 2016). We might then argue that psychoanalysis’ necessarily unproductive mode of care, and its remarkable survival in conditions that would suggest that it is utterly redundant, can be suggestive of a different set of social relations based on a currency of repetitive time that produces care. Clinical listening is precisely the capacity to come back again and again to hear something once more. As I argued above, this repetitive form of care also necessitates a recursive reflexivity within psychoanalytic theory and practice that undoes its disavowed reliance on forms of white privilege. Only then can this idea of mutual aid – of psychoanalysis and ‘the people’ having a relation to one another – be re-established in the now, so that time and care can both circulate.

## Aquilombamento *as Care of the Possible*

Isabelle Stengers’s notion of ‘care of the possible’ (Stengers, 2015) is pertinent here. Stengers’ search for pragmatic ways of living on in the wake of ongoing global catastrophes entails holding open and taking care of possible futures that are distinct from the present. To do this she appeals to what she calls a ‘pharmacological approach’. In an attempt to discern in the present what may make the future, a pharmacological approach pays attention to ‘possibles’ which are neither positive nor negative, where gift and poison are hard to distinguish, and where the effects of experiments – social, political, clinical and artistic, as much as scientific – cannot be known about in advance. Such experiments in care entail suspending preconceived ideas about what care is, what it is able to be, and what it can do, as well as how care is distributed, received, shared, and withheld. Whether care ‘forms’ as a psychoanalytic exchange, as a new psychoanalytic economy between time, money, and care, or as a poem, a letter, a fragment of remembered music, or as a meaningful sign that produces a formal ethics of care, one way of attending to their potential as ‘possibles’ might be to *wait* in the suspended time of their formation, as they take possession of their own logic for disciplining the care that they make.

In 2020, as care was resurfacing from the feminised and racialised margins where it is traditionally relegated, to the top of political and activist agendas, a collection of letters was published by the New Alphabet School based at the arts centre HKV, Berlin, which instigated a conversation between the American philosopher of care ethics, Joan Tronto, and a number of artists, activists, and academics (Karjevsky et al., 2020). The editors’ interest in instigating the exchange of letters was to make a move from thinking about care’s *institutions* – the focus of earlier generations of feminists in their work to unsettle, queer, and reclaim sites of social reproduction such as the ‘family’, and to politicise care in the context of neoliberal democracies that narrowly define the state as the market, and citizens as those who work for and benefit from the market – to care as *infrastructure*. If the infrastructure of state violence entails the broader structural conditions of ‘social, racial and gender injustices that are products of systematic and infrastructural forms that constantly reproduce the same mechanisms of exclusion and marginalisation’ (Karjevsky *et al*., 2020, p. 3), then care as infrastructure would seek to know about and intervene in these conditions. Care as infrastructure rather than institution would, they write, entail maintenance and repair in the place of innovation and growth, recuperation instead of discovery and extraction, and practices that entail accepting limits rather than championing expansion.

What emerges in the exchange of letters is a series of junctures between Tronto’s work, and voices that speak directly from the infrastructure. Tronto understands care as social practice, value, and disposition, instrumentalised through the tasks of caring about, taking care of, care-giving, care-receiving, and caring-with. It is an ethic that can be used to democratize the power of state institutions through the insistence that they take responsibility for care rather than delegating care to largely unpaid racialised and feminized ‘others’. The letters on the other hand, draw on long histories of Black feminist thought, queer theory, disability activism, and work emerging from the ecological crisis, to animate care that takes the form of maintenance, repair, recuperation, and limit. Yayra Sumah, for instance, talks about the ‘work before the work’ of care (Sumah, 2020, p. 47), the work of self-care that can find paths out of the traumas of living under the histories and conditions of western modernity (Sumah, 2020, p. 47). As well as self-care, this ‘work before the work’ needs, in Sumah’s view, to return to difficult questions about the difference between care and love as they play out in the scene of mothering, as it is here that care and vulnerability are constantly at risk of opening onto violence, abuse, and domination, rendering care ambivalent in the infrastructures of violence, at best. In the absence of ‘an organized politics aimed at ending imperialism, white supremacy, capitalism and patriarchy, care work will continue to co-exist with, and facilitate oppression’ (p. 47). Similarly, Edna Bonhomme, drawing on Claudia Rankine’s work, writes of the emotional burden of witnessing on a daily basis Black death. The exploitation of Black life, however, she argues, also evokes a Black radical reimagination of life and care (Bonhomme, 2020, p. 77). ‘Radical care is a tapestry invoking Black feminism, Black radical tradition, and creativity’ (p. 77). Through the labour of careful yet exhausting dismantling of the infrastructures of racism, Black radical care shifts the focus from suffering to freedom. In this sense, Bonhomme writes, ‘care is a fight for full abolition’ (p. 78). And the disability activist Johanna Hedva, writing to Joan Tronto about illness, also reconfigures care’s revolutionary potential through the embrace of exhaustion and depletion as collective and shared responses to the realities of the interdependencies and vulnerabilities that everyone is caught in, yet are projected into the ill or dis-abled body:

Now might be a good time to rethink what a revolution can look like. Perhaps it doesn’t look like a march of angry, abled bodies in the streets. Perhaps it looks something more like the world standing still because all the bodies in it are exhausted – because care has to be prioritized before it’s too late. (Hedva, 2020, p. 70).

Care as the ‘work before the work’, as an ‘abolitionist gesture’ and as the still-standing of the collective exhausted sick body, all suspend the question of what care ‘is’, what it is able to be, and what it can do, as well as how care is distributed, received, shared, and withheld. Instead, it orientates us towards care as something ‘in the making’ of lives that are worth living.

Care, then, is not so much an action but a temporal form, in the sense of the suspension of time that knowingly aims not so much at an alternative future but at a present that will not and cannot budge. The ‘fight’ for full abolition from the position of infrastructure cannot take place in a time of already achieved health and well-being: the future time of a cared-for body and world, or the imagined psychic time before or beyond trauma. Nor can it take place from the perspective of a future in which ‘nature’ has returned to its background position after ‘we’ collectively stave off the current ecological crisis through renewed technological innovation and development, as Isabelle Stengers argues in *In Catastrophic Times* (Stengers, 2015). The catastrophic present coexists with normality:

We live in strange times, a little as if we were suspended between two histories, both of which speak of a world become ‘global.’ One of them is familiar to us. It has the rhythm of news from the front in the great worldwide competition and has economic growth for its arrow of time. It has the clarity of evidence with regard to what it requires and promotes, but it is marked by a remarkable confusion as to its consequences. The other, by contrast, could be called distinct with regard to what is in the process of happening, but it is obscure with regard to what it requires, the response to give to what is in the process of happening. (Stengers, 2015, p. 1)

The ‘work before the work’; the ‘abolitionist gesture’; the ‘world standing still’, are the forms of remaining in the impasse in which one knows what is happening, but what is required nevertheless remains obscure. It is this that Stengers’ work aims at: to ‘try to see in the present what perhaps will make the future’ (Stengers, 2010, p. 12). Although, following William James’ version of pragmatism, the present is the condition in which we cannot know the consequences of practices, we can nevertheless learn to examine situations from the point of view of their possibilities, ‘from that which they communicate with and that which they poison. Pragmatism is the care of the possible’ (p. 12). Care of the possible cannot foreclose on the effects of experiments – and all thoughts and actions are experimentation from this perspective. The effects may be positive or negative, gift or poison. But understanding in what she calls a ‘consequent’ mode means remaining both in contact with the ways in which practices have been destroyed, poisoned and enslaved, and yet remain open to the possibilities for transformation.

What kind of form, then, does care take in a ‘consequent’ mode? If a consequent mode has something to do with a kind of pragmatism that refuses to foreclose on the effects of experimentation, that focuses, in Stengers terms, on ‘adventure’ rather than ‘conquest’ (Stengers, 2017, pp. 144-145) and refrains from doing violence to the ways and worlds of others, then it has a relation to an awareness of what has happened, is happening and will happen simultaneously. In Another Science is Possible: Manifesto for a Slow Science (2017), Stengers calls for the possibility of hesitation, of *ralentissement*, which is the possibility of thought. This hesitation I would call ‘waiting’.

Such waiting could be said to be ‘informal’ time, in the sense of the mid-fifteenth century meaning of informal as ‘lacking form; not in accordance with the rules of formal logic’, before informal takes on its more colloquial meaning of ‘irregular, unofficial’, not according to rule or custom’ or later still, ‘done without ceremony’ (OED). Lacking form is not against form, but lacking the rules of formal logic, presenting as another form of form. Cecil Balmond, the Sri Lankan-British designer, engineer, and artist talks of the ‘informal’ as a practice of ‘compiling an interval’ rather than ‘spacing a gap’. Building the interval up ‘from an inner logic is better than calculating what space should go between elements’ (Balmond, 2007, p. 122). Here the informal creates varying rhythms and wayward impulses instead of regular, formally controlled measures that create structure in the architectural sense. The informal is free-form, constituting the non-linear characteristics of design (p. 113) – a way of going astray as a non-purposeful way to bring about something new. Bronislaw Szerszynski calls this ‘drift’, non-purposeful forms of action that are the form of varied ‘life’ processes (Szerszynski, 2019). From a deep adaptationist perspective, for example, it no longer makes sense to ‘save’ the climate. Instead, we have to give up control and forms of life associated with control. To get off the growth machine that is driving ecological disaster there is some efficacy in stopping being more efficient – insulating more, or developing more and more renewables – and instead learn from systems whose form is ‘drift’, to learn how such forms cope with excess, and hence how to understand inaction differently. Waiting becomes a complex thing to do.

Psychoanalysis has its own lexicon for ‘drift’ that may include evenly-suspended attention, reverie, negative capability, listening without memory or desire, all of which we could term ‘clinical listening’ that we have been discussing here. I would argue that revolutionary time embraced by the psychoanalytic social clinics elaborated in this special issue of *Psychoanalysis and History* gives rise to care through psychoanalytic and psychosocial practices that take care of the possible, in Stengers’ terms. They enact care, in other words, through hesitation, through listening with no outcome in mind, and through the elongated waiting entailed in working through.

To add to the list of psychoanalytic terms for drift, I want to close by drawing out one more, offered by Kwame Yonatan in his important paper titled ‘*Aquilombamento nas Margens*: A Clinic and the Struggle for Existence’. Here he describes the clinical ‘device’, *Aquilombamento nas Margen*, used by the psychoanalysts and psychologists in the *Margens Clínicas* group to address the question of how psychoanalysis can confront state violence. Quilombo, Yonatan explains, is a world without colonial walls. Given that colonization is a wound that still has not healed, quilombo is an ongoing place of refuge and healing where a different reality can be at least imaginatively sustained, without this reality having to be elaborated or detailed as such. The word derives from languages brought to Brazil by enslaved Africans who then escaped slavery and established places of safety in rural communities prior to abolition. At least 3,500 of those original rural quilombos still exist. However, Yonatan elaborates how quilombo as a term now takes on a wider meaning, one that is less tied to physical place, and can be used to describe any inventive times and spaces without colonial walls, where Black people can meet as equals and look after one another. A quilombo can refer to a person, a literary text, an historical period, an imagined future, a poem, a dream. The term can take the form of a verb: to aquilombar on social media, in parliament, in music, art, or literature, is to make that space-time available, to take care of its possibles. I understand ‘aquilombamento’ as the act of coming together in the name of anti-racist practice, in order to defend the rights of Black people, poor people, LGBTQ people, and to demand state actions that protect and care for all of its citizens. But as a psychoanalytic practice, I would suggest that *aquilombamento nas margen* is precisely a form of care of the possible – a way to hold open a place of safety in the name of a future without oppression – but without knowing in advance that it will happen. The clinical listening that each of the projects discussed here offers, is, I think a way to take care of the possible, socially, politically, and psychically.

## Footnotes

1 See https://waitingtimes.exeter.ac.uk/

3 See Introduction in this issue.
